# Jumping Joints: The Complex Relationship Between Osteoarthritis and Jumping Mechanography

**DOI:** 10.1007/s00223-019-00622-0

**Published:** 2019-10-26

**Authors:** C. Shere, N. R. Fuggle, M. H. Edward, C. M. Parsons, K. A. Jameson, C. Cooper, E. M. Dennison, K. A. Ward

**Affiliations:** 1 MRC Lifecourse Epidemiology Unit, University of Southampton, Southampton General Hospital, Tremona Road, Southampton, SO16 6YD UK; 2 Portsmouth Hospitals NHS Trust, Portsmouth, UK; 3 National Institute for Health Research Biomedical Research Centre, University of Southampton and University Hospital Southampton NHS Foundation Trust, Southampton, UK; 4 National Institute for Health Research Musculoskeletal Biomedical Research Unit, University of Oxford, Oxford, UK; 5 MRC Nutrition and Bone Health Research Group, Cambridge, UK

**Keywords:** Osteoarthritis, Jumping mechanography, Muscle, Sarcopenia, Aging

## Abstract

We investigated the relationship between lower limb osteoarthritis (OA) and muscle strength and power (assessed by jumping mechanography) in UK community-dwelling older adults. We recruited 249 older adults (144 males, 105 females). OA was assessed clinically at the knee according to ACR criteria and radiographically, at the knee and hip, using Kellgren and Lawrence grading. Two-footed jumping tests were performed using a Leonardo Mechanography Ground Reaction Force Platform to assess maximum muscle force, power and Esslinger Fitness Index. Linear regression was used to assess the relationship between OA and jumping outcomes. Results are presented as *β* (95% confidence interval). The mean age of participants was 75.2 years (SD 2.6). Males had a significantly higher maximum relative power during lift off (mean 25.7 W/kg vs. 19.9 W/kg) and maximum total force during lift off (mean 21.0 N/kg vs. 19.1 N/kg) than females. In adjusted models, we found significant associations in males between clinical knee OA and maximum relative power [− 6.00 (CI − 9.10, − 2.94)] and Esslinger Fitness Index [− 19.3 (− 29.0, − 9.7)]. In females, radiographic knee OA was associated with total maximum power [− 2.0 (− 3.9, − 0.1)] and Esslinger Fitness Index [− 8.2 (− 15.9, − 0.4)]. No significant associations were observed for maximum total force. We observed significant negative associations between maximum relative power and Esslinger Fitness Index and clinical knee OA in males and radiographic knee OA in females. We have used novel methodology to demonstrate relationships between muscle function and OA in older adults.

## Introduction

Osteoarthritis is the most prevalent chronic joint condition, resulting in pain, loss of function, and reduced quality of life. An estimated 27 million US and 8.5 million UK adults suffer from osteoarthritis, and it is ranked the 11th highest contributor to disability worldwide [1].

Once thought of as a classically degenerative disease of wear and tear, interest is growing in a more systemic pathogenesis, with inflammatory and metabolic components. It is recognised as a disease of the whole joint, involving articular cartilage, bone, ligaments, peri-articular soft tissue and muscle [2].

Muscle weakness is a recognised feature of osteoarthritis, with reports of 20–40% weaker quadriceps in knee osteoarthritis patients compared with age-matched controls [3]. Proposed mechanisms behind this relationship include the role of these muscles in stabilising the joint and as shock absorbers, to reduce joint loading and muscle weakness is also associated with worsening pain and physical function [4]. Similarly, hip osteoarthritis is associated with generalised lower limb muscle weakness [5]. The importance of maintaining good muscle function in osteoarthritis is reflected in recommendations for strengthening exercises in clinical guidelines [6–8].

Jumping mechanography is a novel technique to assess functional muscle parameters from a one- or two-footed jump. Jumping is a complex movement which incorporates muscle function as well as coordination of muscle groups and integration of sensory inputs including proprioception and the vestibular system. It requires high-intensity muscle function, enabling the measurement of peak muscle capacity in performing a real-life action against gravity, rather than more artificial measures of muscle function such as isometric muscle strength. It is, therefore, potentially a measure of more global muscle function, and thus, more representative of everyday life. Jumping mechanography is a validated tool, which has shown correlation with traditional measures of muscle and functional capacity, with good reproducibility, little learning effect, and good safety [9]. Additionally, it provides novel insights into lower limb muscle power and force which take the understanding of muscle physiology beyond that provided by muscle strength alone. Studies have shown that jumping mechanography is able to detect age-related declines in muscle capability earlier than traditional measures of physical capability, suggesting it may be a more sensitive tool to detect functional muscle deterioration [10, 11], however, data describing the relationship between jumping mechanography and osteoarthritis is lacking.

For this reason, the aim of this study was to explore the relationship between whether muscle function, assessed using jumping mechanography, differed between participants with clinical or radiographic lower limb osteoarthritis compared to those without and investigate the relationship between continuous variables and jumping mechanography outcomes.

## Methods

Participants were members of the Hertfordshire Cohort Study (HCS), a large prospective study of the lifecourse origins of disease involving community-dwelling men and women born in Hertfordshire between 1931 and 1939. The HCS has previously been described and is generally representative of the UK population as a whole [12, 13]. Of the 376 participants enrolled, 249 were able or willing to perform jumping mechanography with other exclusions including refusal due to pain or frailty, and the presence of hip or knee arthroplasty.

Osteoarthritis data were obtained from HCS members as part of the UK component of the European Project on Osteoarthritis (EPOSA) [14]. A questionnaire was used to collect demographic information such as smoking status, alcohol intake, physical activity (recorded as minutes per day) and the Western Ontario and McMaster Universities Osteoarthritis Index (WOMAC)—a 24-item questionnaire with 3 subscales measuring pain, stiffness and physical function [15]. Five-point Likert scales ranging from 0 to 4, with 0 indicating none, were used to record WOMAC scores, with a score ≥ 1 indicating symptoms. Trained research nurses collected measurements of height and weight. Clinical examination of the knees was performed and osteoarthritis was diagnosed according to the American College of Rheumatology criteria [16].

Antero-posterior (AP) and lateral knee radiographs were taken of both knees and hips, and graded for osteoarthritis by rheumatologists based on Kellgren and Lawrence score (K&L) scores [17]. Radiographic knee or hip osteoarthritis was defined as a K&L score of ≥ 2. Knee and hip replacements were excluded.

Jumping mechanography assessed a two-footed countermovement jump using a Leonardo Mechanography Ground Reaction Force Platform (Leonardo software version 4.2; Novotec Medical GmbH), to assess lower limb muscle force and power. Study participants were asked to stand on the ground reaction force platform, bend their knees and jump as high as possible. This was repeated 3 times and the highest jump was used to calculate force, power and the Esslinger Fitness Index (EFI); force and power were normalised to body weight (N/kg and W/kg respectively). To clarify, force is equal to mass multiplied by acceleration (*F *= *ma*) and power is equal to force multiplied by velocity (*P* = *Fv*). The EFI compares the relative power per kg body weight of a study participant to the average of an age and sex-matched reference group, and is expressed as a percentage where 100% represents the 50th percentile for sex and age.

### Statistical Methods

Statistical analyses were performed using Stata version 15. Participant characteristics were described using means and standard deviations (SD) for continuous normally distributed variables or median and inter-quartile range (IQR) for skewed continuous variables. Frequencies and percentages were used for binary and categorical variables. To assess for differential effects between males and females, participants were stratified by sex and sex differences were analysed using the *t* test, Wilcoxon-ranksum test, Chi squared or Fisher’s exact tests.

Linear regression analysis was used to investigate associations between jumping mechanography data and clinical and radiographic osteoarthritis, unadjusted and adjusted for age, height, social class, smoker status, alcohol consumption, physical activity and pain in the corresponding joint (when examining associations with radiographic osteoarthritis, using the corresponding continuous WOMAC scale). Results of the regression analyses are presented as regression coefficients (*β*) and 95% confidence intervals (95% CI).

Males and females were analysed separated and thus sex was not included as an adjustment in any model used. EFI is adjusted for sex and age against a reference population and so age was excluded as an adjustment from EFI statistical models.

Statistical significance was set at 5%.

## Results

### Demographics

The mean age of participants was 75.2 (SD 2.6) years for both males and females (Table 1). Males were significantly heavier [mean 82.4 kg (SD 11.9) vs. 70.1 kg (SD 11.6), *p *< 0.001] and taller [mean 173.0 cm (SD 6.2) vs. 159.1 cm (SD 5.5), *p* < 0.01] than females, though there was no significant difference in BMI (*p *= 0.81). There were no significant sex differences in the prevalence of clinical or radiographic osteoarthritis at the knee [males 11 (7.6%) clinical, 60 (43.2%) radiographic; females 12 (11.4%) clinical, 49 (50.5%) radiographic] or radiographic osteoarthritis of the hip [males 67 (48.2%); females 37 (39.8%)]. Males consumed more alcohol (median 7.2 units/week vs. 1.5 units/week, *p *< 0.001) and were more likely to have ever smoked (*p *< 0.02).

**Table 1:** Participant characteristics

Mean (SD)	Males (*n* = 144)	Females (*n *= 105)	*p* value
Age (years)	75.2 (2.4)	75.2 (2.6)	0.900
Height (cm)	173.0 (6.2)	159.1 (5.5)	**< 0.001**
Weight (kg)	82.4 (11.9)	70.1 (11.6)	**< 0.001**
BMI (kg/m^2^)	27.5 (3.8)	27.7 (4.4)	0.808
Smoking status^d^			
Never	60 (41.7%)	63 (60.0%)	**0.010**
Ex-smoker	78 (54.2%)	37 (35.2%)	
Current smoker	6 (4.2%)	5 (4.8%)	
Alcohol consumption (units/week)*	7.2 (1.9, 14.1)	1.5 (0.1, 4.8)	**< 0.001**
Activity time in last 2 weeks (min/day)*	200 (131–291)	208 (154–285)	0.619
Social class			
I-IIINM	64 (46.4%)	43 (41.0%)	0.399
IIIM-V	74 (53.6%)	62 (59.0%)	
WOMAC score^ad^			
WOMAC knee pain	37 (25.7%)	28 (26.7%)	0.863
WOMAC hip pain	21 (14.6%)	18 (17.5%)	0.539476
Osteoarthritis^d^			
Clinical knee osteoarthritis^b^	11 (7.6%)	12 (11.4%)	0.308
Clinical hip osteoarthritis^b^	2 (1.4%)	5 (4.8%)	0.137
Clinical knee and hip osteoarthritis^b^	0 (0%)	0 (0%)	
Radiographic knee osteoarthritis^bc^	60 (43.2%)	49 (50.5%)	0.265
Radiographic hip osteoarthritis^bc^	67 (48.2%)	37 (39.8%)	0.206
Radiographic knee and hip osteoarthritis^bc^	31 (22.5%)	25 (26.9%)	0.442
Jumping mechanography			
Maximum total power during lift off (kW)	2.10 (0.45)	1.38 (0.31)	**< 0.001**
Maximum total power per body weight (W/kg)	25.7 (5.1)	19.9 (4.4)	**< 0.001**
Maximum total force during lift off (kN)	1.72 (0.30)	1.33 (0.22)	**< 0.001**
Maximum total force per body weight (N/kg)	21.0 (2.8)	19.1 (2.3)	**< 0.001**
Esslinger Fitness Index (EFI)	81.9 (15.8)	84.0 (18.1)	0.323

Significant associations (*p *<0.05) are highlighted in bold

*Median (lower quartile, upper quartile)

^a^WOMAC pain score of 1 plus

^b^Diagnosed by clinical examination using ACR criteria

^c^Kellgren and Lawrence (K&L) grade ≥2

^d^N(%)

Males had a significantly higher maximum relative power [mean 25.7 W/kg (SD 5.1) vs. 19.9 W/kg (SD 4.4), *p *< 0.001] and maximum total force [21.0 N/kg (SD 2.8) vs. 19.1 N/kg (SD 2.3), *p* < 0.001] during lift off than females. There was no significant sex difference for EFI [males 81.9% (SD 15.8) vs. females 84.0% (SD 18.1), *p* = 0.3]. Due to missing data, the total number of participants varied when examining some associations. These are documented in the ‘N’ column for Tables 2, 3 and 4.

**Table 2:** Osteoarthritis as an explanatory variable for maximum relative power during lift off per body weight for males and females, unadjusted and adjusted

	*N*	Regression coefficient	95% CI	*p*-value	*N*	Regression coefficient	95% CI	*p*-value
Males	Unadjusted				Adjusted^a^			
Clinical knee OA	144	− 6.71	(− 9.68, − 3.75)	**< 0.001**	128	− 6.00	(− 9.05, − 2.94)	**< 0.001**

	Unadjusted				Adjusted^b^			
Radiographic knee OA	139	− 1.44	(− 3.14, 0.27)	0.098	123	− 0.76	(− 2.47, 0.96)	0.383
Radiographic hip OA	139	0.16	(− 1.56, 1.87)	0.858	123	− 0.50	(− 2.33, 1.32)	0.587
Radiographic knee & hip OA	139	− 0.06	(− 2.12, 2.00)	0.954	123	− 0.01	(− 2.10, 2.07)	0.989

Females	Unadjusted				Adjusted^a^			
Clinical knee OA	105	− 1.80	(− 4.45, 0.85)	0.180	97	− 2.19	(− 5.17, 0.80)	0.149

	Unadjusted				Adjusted^b^			
Radiographic knee OA	97	− 2.66	(− 4.39, − 0.94)	**0.003**	89	− 2.02	(− 3.89, − 0.14)	**0.035**
Radiographic hip OA	93	0.48	(− 1.42, 2.37)	0.620	84	0.38	(− 1.72, 2.48)	0.719
Radiographic knee & hip OA	95	− 0.30	(− 2.37, 1.78)	0.776	86	− 0.02	(− 2.21, 2.17)	0.986

Bold values indicate *p* < 0.05

^a^Adjusted for age, height, social class, smoker status, alcohol consumption and activity time

^b^Adjusted for age, height, social class, smoker status, alcohol consumption, activity time and pain in the corresponding joint

**Table 3:** Osteoarthritis as an explanatory variable for maximum total Esslinger Fitness Index during lift off per body weight for males and females, unadjusted and adjusted

	*N*	Regression coefficient	95% CI	*p*-value	*N*	Regression coefficient	95% CI	*p*-value
Males	Unadjusted				Adjusted^a^			
Clinical knee OA	144	− 20.7	(− 29.90, − 11.50)	**< 0.001**	128	− 19.33	(− 28.98, − 9.68)	**< 0.001**

	Unadjusted				Adjusted^b^			
Radiographic knee OA	139	− 4.52	(− 9.75, 0.70)	0.089	123	− 2.64	(− 8.06, 2.78)	0.336
Radiographic hip OA	139	0.34	(− 4.91, 5.60)	0.897	123	− 1.38	(− 7.17, 4.40)	0.637
Radiographic knee & hip OA	139	− 0.69	(− 6.99, 5.62)	0.830	123	0.02	(− 6.58, 6.62)	0.995

Females	Unadjusted				Adjusted^a^			
Clinical knee OA	105	− 9.26	(− 20.20, 1.69)	0.097	97	− 9.06	(− 21.30, 3.18)	0.145

	Unadjusted				Adjusted^b^			
Radiographic knee OA	97	− 10.2	(− 17.43, − 2.96)	**0.006**	89	− 8.17	(− 15.91, − 0.42)	**0.039**
Radiographic hip OA	93	2.50	(− 5.39, 10.40)	0.530	84	1.76	(− 6.96, 10.48)	0.689
Radiographic knee & hip OA	95	− 0.63	(− 9.27, 8.01)	0.885	86	− 0.05	(− 9.13, 9.03)	0.991

Bold values indicate *p* < 0.05

^a^Adjusted for height, social class, smoker status, alcohol consumption and activity time

^b^Adjusted for height, social class, smoker status, alcohol consumption, activity time and pain in the corresponding joint

**Table 4:** Osteoarthritis as an explanatory variable for maximum total force during lift off per body weight for males and females, unadjusted and adjusted

	*N*	Regression coefficient	95% CI	*p*-value	*N*	Regression coefficient	95% CI	*p*-value
Males	Unadjusted				Adjusted^a^			
Clinical knee OA	144	− 0.33	(− 2.08, 1.42)	0.710	128	− 0.46	(− 2.26, 1.35)	0.617

	Unadjusted				Adjusted^b^			
Radiographic knee OA	139	− 0.75	(− 1.70, 0.21)	0.125	123	− 0.72	(− 1.74, 0.29)	0.159
Radiographic hip OA	139	0.28	(− 0.67, 1.24)	0.556	123	0.15	(− 0.90, 1.19)	0.784
Radiographic knee & hip OA	139	− 0.30	(− 1.44, 0.85)	0.607	123	− 0.50	(− 1.73, 0.72)	0.416

Females	Unadjusted				Adjusted^a^			
Clinical knee OA	105	− 0.05	(− 1.44, 1.34)	0.948	97	− 0.58	(− 2.17, 1.01)	0.471

	Unadjusted				Adjusted^b^			
Radiographic knee OA	97	− 0.92	(− 1.82, − 0.01)	**0.048**	89	− 0.95	(− 1.97, 0.06)	0.064
Radiographic hip OA	93	− 0.61	(− 1.57, 0.36)	0.214	84	− 0.47	(− 1.53, 0.58)	0.377
Radiographic knee & hip OA	95	− 0.77	(− 1.83, 0.29)	0.154	86	− 0.58	(− 1.74, 0.58)	0.325

Bold value indicates *p* < 0.05

^a^Adjusted for age, height, social class, smoker status, alcohol consumption and activity time

^b^Adjusted for age, height, social class, smoker status, alcohol consumption, activity time and pain in the corresponding joint

### WOMAC Pain Scores

There was no significant difference between males and females for WOMAC knee pain or hip pain (25.7% males and 26.7% female, 14.6% males and 17.5% females; respectively).

### Jumping Mechanography and Osteoarthritis

#### Maximum Relative Power

We found statistically significant negative associations between maximum relative power (W/kg) and clinical knee osteoarthritis in males [β − 6.71 (95% CI − 9.68, − 3.75) *p* < 0.01] and radiographic knee osteoarthritis in females [β − 2.66 (95% CI − 4.39, − 0.94) *p* ≤ 0.01] (Table 2). These associations remained significant after adjustment for age, height, social class, smoker status, alcohol consumption and physical activity and pain in the corresponding joint (for radiographic osteoarthritis).

No significant associations were seen between maximum relative power and clinical knee osteoarthritis in females [*β* − 1.80 (95% CI − 4.45, 0.85) *p* = 0.18] or radiographic knee [*β* − 1.44 (95% CI − 3.14, 0.27) *p *= 0.01] or hip [*β* 0.16 (95% CI − 1.56, 1.87) *p *= 0.86] osteoarthritis in males, or radiographic hip osteoarthritis in females [*β* 0.48 (95% CI − 1.42, 2.37) *p *= 0.62].

#### Esslinger Fitness Index (EFI)

Statistically significant negative associations were observed between EFI and clinical knee osteoarthritis in males [β − 20.7 (95% CI − 29.90, − 11.50) *p *< 0.001] and radiographic knee osteoarthritis in females [*β* − 10.2 (95% CI − 17.43, − 2.96) *p *< 0.01] (Table 3). These associations remained significant after adjustment.

#### Maximum Total Force

We observed a statistically significant negative association between maximum total force (N/kg) and radiographic knee osteoarthritis in females [*β* − 0.92 (95% CI − 1.82, − 0.01) *p *< 0.05], although this was non-significant after adjustment (Table 4). No other statistically significant associations were seen between maximum total force during lift off and osteoarthritis in males or females.

#### Adverse Effects

There were no adverse effects associated with the performance of the two-footed jumping mechanography.

## Discussion

In this population of elderly individuals, we found that maximal muscle power was negatively associated with clinical knee osteoarthritis in males and radiographic knee osteoarthritis in females. Muscle force was associated with radiographic knee osteoarthritis in females, although this was attenuated after adjustment. These findings, highlight the association between osteoarthritis and muscle power rather than force and are in agreement with previous studies [18–20].

The reason for the sex-discrepancy in relationship between osteoarthritis and muscle power could be due to differential pain processing between the sexes or due to elements of the American College of Rheumatology (ACR) criteria for osteoarthritis which affect the sexes differently.

For clarity, the ACR clinical classification criteria for osteoarthritis of the knee use pain in the knee combined with a least three elements of physical examination as detailed; aged > 50 years, < 30 min of morning stiffness, crepitus on active motion, bony tenderness, bony enlargement, no palpable warmth of synovium. Although the above criteria were used in the current study, other ACR clinical classification criteria can include radiographic findings (including osteophytes) or laboratory findings (including normal inflammatory markers, rheumatoid factor or synovial fluid consistent with osteoarthritis).

The beta coefficients observed for EFI were higher than those for relative jumping power. This may be due to a reduction in the variance in EFI values as the index is calculated relative to a age and sex-adjusted population.

The explosive nature of a maximal jump means jumping mechanography assesses the maximal speed of neuromuscular transmission and actin-myosin cross-bridge cycling, reflected in the measurement of muscle power. This is in contrast to measures of maximal force or strength measured using instruments such as dynamometers, where there is unlimited time for recruitment of motor units and optimal engagement of muscle filaments, which may therefore not detect slowing of these components. This slowing is well documented with regards to aging [21], and there is growing evidence of similar processes occurring in osteoarthritis [3]. This reinforces the importance of measures of muscle power which include velocity, to detect early changes in neuromuscular function.

Individuals with knee osteoarthritis have reduced proprioceptive acuity and increased postural sway compared with healthy age- and sex-matched controls, potentially related to mechanoreceptor dysfunction secondary to joint damage [22] which may have impeded the ability to generate jumping power.

In osteoarthritis, the recruitment of larger motor units has been reported, which may represent sprouting of collateral neurones to activate more muscle fibres, in an attempt to compensate for reduced nerve firing rates to preserve contractile ability, which would result in a larger motor unit [23]. A similar process occurs during aging, and is proposed to enable preservation of maximal force, despite reductions in power [21]. Finally, intrinsic muscle properties take centre stage. In osteoarthritis, there is higher thigh muscle fat infiltration, inflammation and pro-fibrotic extracellular matrix, associated with reduced muscle strength [24, 25]. Vastus lateralis muscle biopsies from osteoarthritis patients have shown a reduction in muscle fibre size, especially apparent in fast-twitch type IIa fibres [26], along with reduced power and force production and slower cross-bridge kinetics [27]. There is also evidence of neurogenic muscle atrophy and denervation, in keeping with the arthrogenic muscle inhibition (AMI) mechanism [26]. These findings reflect the complex and interconnected nature of the relationship between muscle weakness and osteoarthritis.

**Fig. 1:**
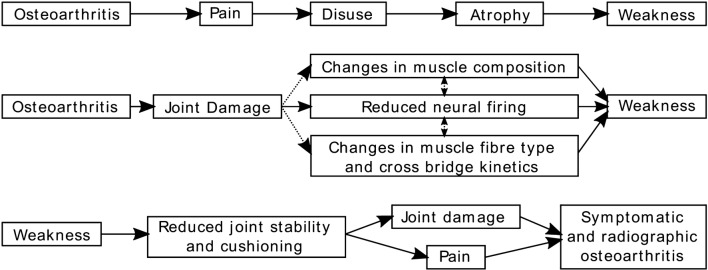
Proposed sequences of events for the relationship between muscle weakness and osteoarthritis, with either osteoarthritis-induced joint damage or pain, or muscle weakness as the initial insult

Inherent to the cross-sectional design of this study, temporal causation cannot be inferred, which brings into question whether reductions in muscle power are a result of osteoarthritic disease, or whether reductions in muscle power predispose to osteoarthritis. Potential sequences of events are shown in Fig. 1. If muscle weakness is a result of osteoarthritis, one explanation is limitation by pain, leading to muscle disuse. However, in this population, WOMAC pain scores were low and the association of reduced muscle power with radiographic knee arthritis in females remained after adjustment for pain, as previously demonstrated [28]. The finding that reduced muscle power is associated with structural joint damage in females, which is not explained by pain, suggests another underlying mechanism rather than muscle disuse. An alternative explanation could be that osteoarthritic joint damage could lead to overstimulation of mechanoreceptors, perhaps in the absence of clinical symptoms, as in the proposed AMI mechanism, eventually leading to reduced neural stimulation of muscle, alterations in muscle innervation, composition, and weakness. This mechanism is supported by the widely reported poor correlation of radiographic osteoarthritis with clinical symptoms, meaning that joint damage can occur in the absence of pain, and vice versa [29, 30], though concordance has also been demonstrated [31].

Conversely, muscle weakness itself may be a risk factor for the development of osteoarthritis. Lower limb muscles stabilise the hip and knee joints, and provide cushioning, especially during phases of the gait which put mechanical stress on the joint, acting as a shock absorber [32]. Therefore, muscle weakness could lead to excessive stress and abnormal loading on the joint, predisposing to the development of osteoarthritis. Several longitudinal studies have found that quadriceps muscle weakness increases the risk of developing radiographic knee osteoarthritis, usually only in females, which the authors attribute to females having a lower baseline muscle strength, so perturbations may lower strength below a threshold for developing osteoarthritis [33–35]. Indeed, a systematic review found knee extensor weakness conferred a 1.65-fold greater risk of developing symptomatic knee osteoarthritis, and a 1.85-fold greater risk of developing radiographic knee osteoarthritis [36]. Overall, these findings warrant further longitudinal investigation to elucidate the temporal relationship between quadriceps muscle weakness and osteoarthritis.

The strengths of this study include the use of jumping mechanography, as a novel tool to provide a dynamic functional representation of muscle capability in osteoarthritis, incorporating multiple sensory systems. Additionally, the Hertfordshire Cohort Study represents a detailed characterisation of community-dwelling older adults in the United Kingdom and measurements were carried out according to strict protocols by trained, epidemiological fieldworkers. Previous work has demonstrated that the cohort are representative of the wider UK population [12].

Potential limitations of this study include a possible healthy bias, as only those that were able or willing to jump could be included in the study. There was a relatively low prevalence of clinical hip osteoarthritis (2 males and 5 females) preventing us from studying osteoarthritis at this site. Significant associations between female clinical hip osteoarthritis and relative jumping power and Esslinger fitness index were found and a similar, non-significant trend was observed in males with osteoarthritis, however, (due to the low prevalence) these data are not shown.

In terms of the application of our findings, we have described the cross-sectional associations of jumping mechanography with knee and hip osteoarthritis in this cohort. If the clinical utility of jumping mechanography is to be tested and utilised, it is crucial that associations in other populations are investigated and longitudinal relationships explored. Muscle strengthening exercise is already recommended in clinical guidelines worldwide [6–8], based on improvements in pain and function [37]. In people with osteoarthritis, intervention studies have found better functional outcomes for high-velocity versus low-velocity training [38]. For clinical practice, our findings therefore support the benefit of physical exercise regimes which promote greater muscle power in osteoarthritis, namely high-velocity resistance training.

## Conclusions

We have used a novel technique, jumping mechanography, to describe associations between the ability of muscles to generate force rapidly and clinical knee osteoarthritis in males and radiographic knee osteoarthritis in females. This highlights the potential role of muscle weakness in osteoarthritis, specifically the ability of muscle to generate force rapidly, represented by peak muscle power. Further investigation into muscle weakness as an aetiology or as a result of osteoarthritis are warranted, through longitudinal studies. These findings have clinical implications, suggesting high-velocity resistance training may be particularly beneficial in people with osteoarthritis, to improve muscle power.
